# Simulation study on dynamic characteristics of gas diffusion in coal under nitrogen injection

**DOI:** 10.1038/s41598-022-23778-6

**Published:** 2022-11-07

**Authors:** Xinliang Fang

**Affiliations:** School of Emergency Management and Safety Engineering, China University of Mining and Technology, Beijing, 100083 China

**Keywords:** Energy, Environmental chemistry, Surface chemistry

## Abstract

To reveal the mechanism of desorption of methane in coal seams by inert gas N_2_, the desorption behavior of CH_4_ after N_2_ injection was studied by using Giant Canonical ensemble Monte Carlo (GCMC) and Molecular Dynamics (MD) methods with wiser bituminous coal as the research object. The results show that the adsorption isotherms of CH_4_ and N_2_ in the molecular structure model of bituminous coal are in good agreement with the Langmuir adsorption isotherm model. The adsorption capacity of the two gases in the bituminous coal structure model is CH_4_ > N_2_. The higher the N_2_ injection pressure, the higher the temperature, and the more methane desorption. N_2_ can replace some adsorbed CH_4_ through competitive adsorption with CH_4_. Compared with injecting high-temperature nitrogen to desorb methane in coal seams, in high-pressure nitrogen, the diffusion effect of CH_4_ flowing in coal is more significant. The higher the nitrogen injection pressure, the better the effect of N_2_ promoting CH_4_ desorption. The relative concentration of CH_4_ in the vacuum layer gradually increases with the increase of water content. This indicates that the water in coal promotes the desorption of CH_4_. The mechanism of N_2_ injection and CH_4_ desorption in coal seams mainly includes gas displacement and gas dilution and diffusion. This study provides theoretical support for methane extraction technology in goaf.

## Introduction

Gas injection mining is an important stimulation technology for CBM development. For coal seams with poor gas permeability, low permeability, and rapid borehole gas flow decay, traditional negative pressure drainage takes a long time and achieves slow results, which can no longer meet the needs of mining replacement and underground safety production^1–3^. Coal seam gas injection not only increases the internal pressure of the coal body and increases the gas seepage velocity, but also reduces the effective partial pressure of gas and promotes the desorption of adsorbed gas^4,5^. Therefore, it is of great significance to study CBM gas injection stimulation technology and improve the permeability of CBM wells.

Many scholars have carried out relevant studies on nitrogen injection to promote emission/gas extraction. Clarkson et al.^6^ believed that injecting non-gas gas into coal seam can reduce the partial pressure of the gas, promote the desorption of adsorbed gas, and increase the gas recovery rate. Yang conducted a field test of low-pressure nitrogen injection in coal seam to promote gas extraction/drainage in the underground coal mine of Yangquan Mining area^7^, and found that injecting nitrogen into coal seam could significantly promote gas extraction/drainage. Katayama^8^ believed that the mechanism of CO_2_ replacing CH_4_ was due to the stronger adsorption capacity of CO_2_, while N_2_ replacing CH_4_ was due to the reduction of the partial pressure of CH_4_ by N_2_ injection. Wu et al.^9^ carried out an experimental study on the effect of nitrogen injection based on the theory of diffusion seepage and multi-component adsorption equilibrium and discussed the stimulation mechanism of gas displaced by nitrogen injection. Most of the above studies are carried out through laboratory experiments and field tests. However, coal is composed of inorganic and organic matter, and its inner surface has complex structural characteristics, so the gas injection displacement mechanism should be explained from the molecular level^10–12^. Molecular simulation method can be used to study the microscopic mechanism of interaction between adsorbent and adsorbent at the molecular level^13,14^.

Scholars at home and abroad have done a lot of research on molecular simulation of the adsorption characteristics of CO_2_, N_2_, and other mixed gases of coalbed methane^15,16^. Wu et al.^17^ used the grand canonical integrated Monte Carlo method to analyze the ability and competitive difference of coal to adsorb CO_2_, O_2_, and N_2_ according to the fire prevention and storage practice of flue gas injected into the goaf of power plant. Lou et al.^18^ established a physical adsorption model for different gas molecules such as O_2_, CO_2_, and N_2_ on the surface of coal macromolecules to explore the gas competition difference of mixed gas molecules on the surface of coal macromolecules. Cui et al.^19^ studied the adsorption of CH_4_, N_2_, and CO_2_ gases by coal, and the results showed that the adsorption capacity of coal for the three gases was CO_2_ > CH_4_ > N_2_. Song et al.^20^ used GCMC and DFT methods to study the influence of oxygen-containing functional groups and electrostatic interaction on the competitive adsorption of CO_2_, CH_4_, and N_2_ in the low-rank coal molecular model, and found that the strong quadrupole moment and polarization ability of CO_2_ led to the stronger selective adsorption of oxygen-containing functional groups than CH_4_ and N_2_.

In terms of nitrogen injection to promote methane extraction, the adsorption/desorption mechanism of different gases in coal is studied mainly through laboratory experiments, field tests, and numerical simulation. In fact, coal itself contains adsorbed methane, and different temperatures, types of injected gas, and injection pressure of coal seam have a great influence on the desorption of coal seam methane. Therefore, the interaction mechanism between injected gas molecules and methane-containing coal in the process of gas injection is not clear at present. Coal contains methane, and the molecular dynamics mechanism of gas injection under different conditions to promote methane desorption in coal needs to be further studied. Although some scholars have studied the mechanism of methane adsorption by coal from a microscopic point of view, there is still a lack of systematic simulation of the influence of water on methane desorption by coal. Therefore, the mechanism of nitrogen injection promoting gas desorption by water in coal has not been fully grasped. Taking Wiser bituminous coal as an example, the molecular simulation was used to study the desorption behavior of CH_4_ in methane-containing coal structure after N_2_ injection at different temperatures, pressures, and moisture content, to clarify the mechanism of promoting CH_4_ desorption in coal by N_2_ injection. It provides theoretical support for methane extraction technology in goaf.

## Calculation methods

### Coal molecular structure model

**Figure 1:**
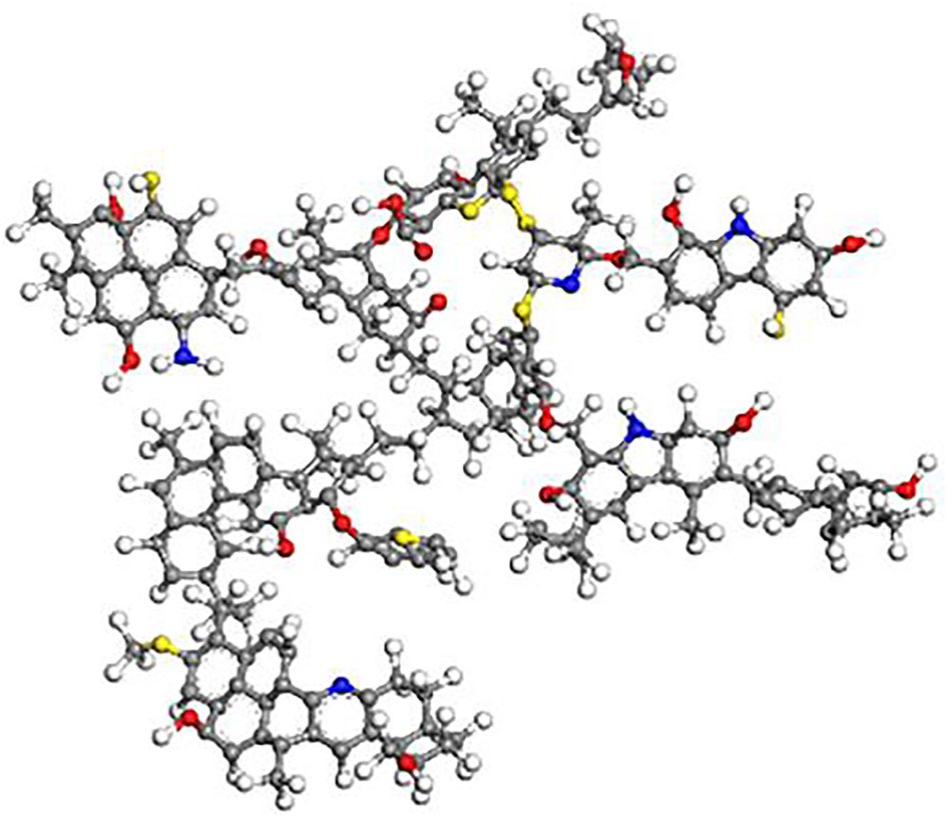
Wiser bituminous coal molecular model.

Theoretical coal models are generally composed of coal molecules consisting of a large number of atoms, and these idealized coal models are applied to solve practical problems^21,22^. In this study, a typical bituminous coal Wiser molecular substrate (C_192_H_165_O_20_N_5_S_8_) was selected, as shown in Fig. 1. The lignite substrate model is composed of an aromatic skeleton amorphous molecular structure composed of 390 atoms, including carbon, hydrogen, oxygen, nitrogen, and sulfur elements. This molecular model has been successfully applied in the study of gas competitive adsorption on bituminous coal surface^23^. Table 1 shows the structural parameters of Wiser bituminous coal macromolecular model.

**Table 1:** Structural parameters of the Wiser bituminous coal macromolecular model.

Molecular formula	Molecular weight	Element content (%)				
		C	H	O	N	S
C_192_H_165_O_20_N_5_S_8_	3115	73.96	10.27	5.30	2.25	8.22

### Molecular dynamics simulation

**Figure 2:**
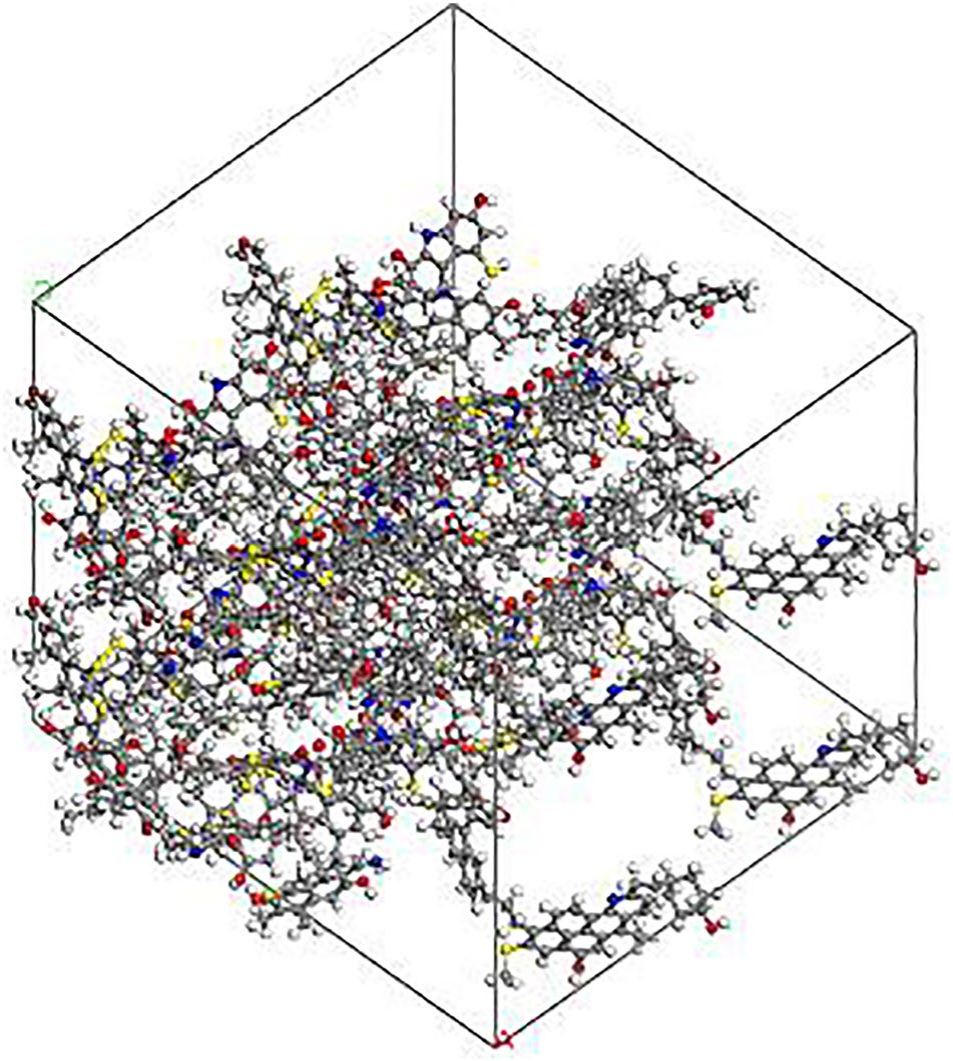
Surface model of bituminous coal.

The molecular dynamics simulation was carried out on Materials Studio software, and the COMPASSII force field was used in the calculation process. In this paper, the surface model of bituminous coal was constructed through the Amorphous Cell module, and the initial surface model of bituminous coal was obtained by filling the 16 optimized bituminous coal molecular models into the 3D periodic boundary Cell. Then Anneal is annealed through Anneal task in Forcite module, the precision is set as Fine, the temperature range is 300–500 K, and the total simulation time is 1 ns. Finally, the structure was further optimized with Fine precision and the Ewald summation method was used for van der Waals interaction with A truncation radius of 15.0 Å to obtain the final bituminous coal surface model (Fig. 2).

**Figure 3:**
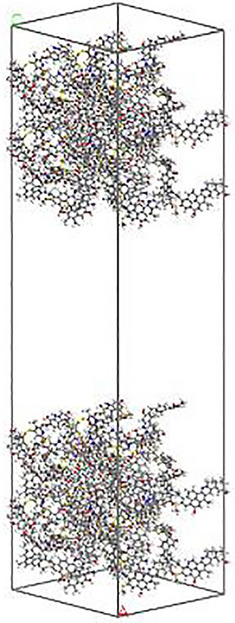
Bituminous coal supercell model.

The density of the model was 1.20 g/cm^3^. The cell was expanded to construct a 1 × 2 × 2 supercell, and 50 Å vacuum layer was added, as shown in Fig. 3.

CH_4_ and N_2_ molecules were adsorbed in the coal surface model to form the CH_4_-N_2_ system, and the structure of the system was optimized by repeating the above structural optimization method. The Dynamics properties are calculated under the Dynamics task in the Forcite module. The NVT canonical ensemble was used to study the dynamics of the system with 40 ps of simulated data. The setting of the calculation method is consistent with that in the structural optimization of the model mentioned above.

Mean Square Displacement (MSD) is the statistical average of particle trajectories, which refers to the measurement of the average distance of other particles around a given particle^24^. MSD calculation formula is as follows:

1 $$  {\text{MSD}} = \frac{1}{{\text{N}}}\left\langle {\sum\limits_{{{\text{i}} = 1}}^{{\text{N}}} {\left[ {{\text{r}}_{{\text{i}}} ({\text{t}}) - {\text{r}}_{{\text{i}}} (0)} \right]^{2} } } \right\rangle   $$

where ${\text{r}}_{{\text{i}}} ({\text{t}})$ and ${\text{r}}_{{\text{i}}} (0)$ are the instantaneous and initial position vectors of the ith particle, respectively. Moreover, N is the total number of particles in the system.

The diffusion coefficient of gas can be obtained from the following expression:

2 $$ {\text{D}} = \frac{1}{{6{\text{N}}}}\mathop {\lim }\limits_{{{\text{t}} \to \infty }} \frac{{\text{d}}}{{{\text{d}}_{{\text{t}}} }}\left\langle\sum \limits_{{{\text{i}} = 1}}^{{\text{N}}} \left[ {{\text{r}}_{{\text{i}}} ({\text{t}}) - {\text{r}}_{{\text{i}}} (0)} \right]^{2}\right\rangle $$

3 $$ {\text{D}} = \mathop {\lim }\limits_{{{\text{t}} \to \infty }} \left( {\frac{{{\text{MSD}}}}{{6{\text{t}}}}} \right) = \frac{1}{6}{\text{K}}_{{{\text{MSD}}}} $$

where D is the diffusion coefficient of the gas, N is the number of particles in the system, and K_MSD_ is the slope of the MSD curve.

### Monte Carlo simulation

In the Sorption module, the injection pressure is 10–1000 kpa, and the temperature is 293–333 K; The number of simulated loading steps to the equilibrium state is 1 × 10^8^ steps, and the total number of steps is 2 × 10^8^ steps. The Metropolis method was chosen for configuration calculation, and the charge balance (QEq) method was used for charge calculation. The Settings of force field, van der Waals force, hydrogen bond force, and Coulomb force calculation methods were consistent with those in the structural optimization of the previous model.

**Figure 4:**
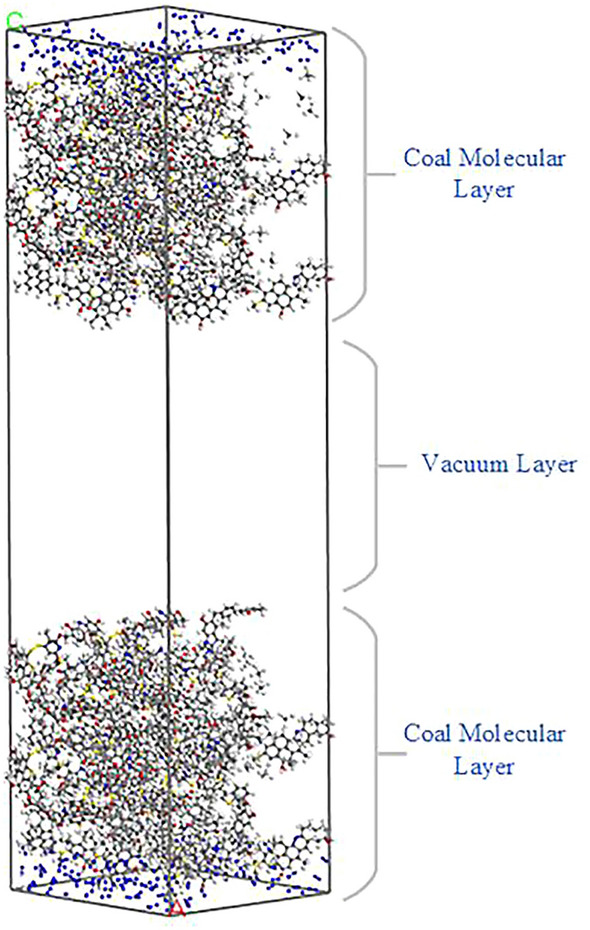
Initial model of nitrogen injection.

Figure 4 shows the initial model of nitrogen injection. Regions 0–57 Å and 107–164 Å are the molecular layers of coal, and region 57–107 Å is the vacuum layer. The amount of N_2_ injected under different pressures is consistent with the adsorption amount of N_2_ in Table 1. The quantity of N_2_ injected at different temperatures was 256. The simulation calculation of desorption CH_4_ adopts the Focite module. The task is set as Dynamics and the temperature is set as 293–333 K. The temperature control method, ensemble and force field are consistent with the Dynamics parameters in “Molecular dynamics simulation” section.

**Table 2:** The quantity of N_2_ injected at different pressures.

Pressure (MPa)	Adsorption amount/n
1	177
2	210
3	279
4	361
5	445

## Results and analysis

### Distribution characteristics of the gas in the model

**Figure 5:**
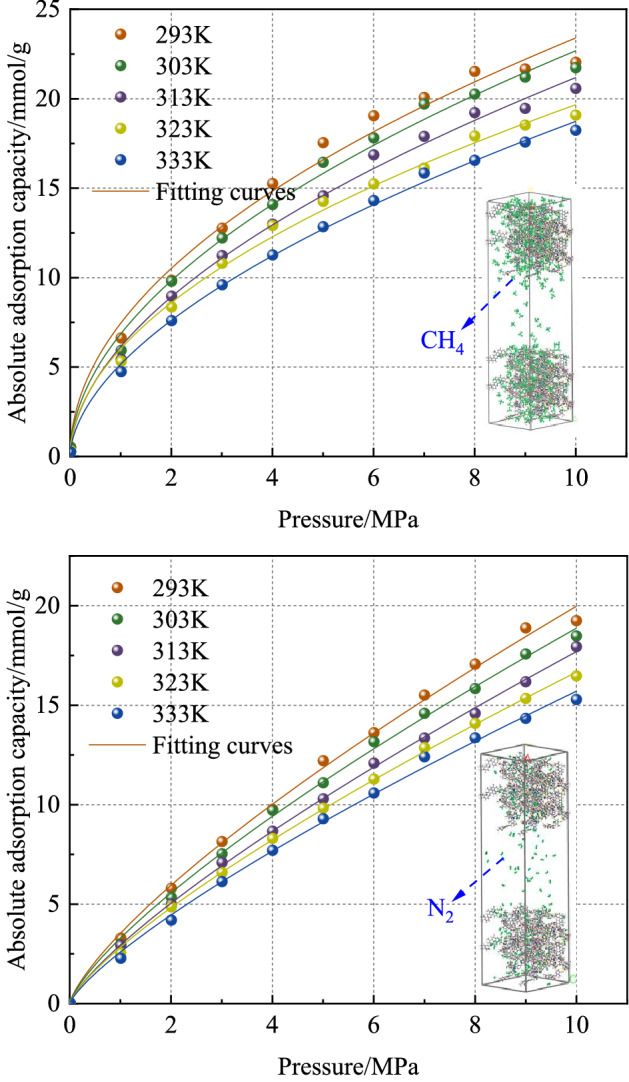
Adsorption isotherms and Langmuir fitting curves of CH_4_ and N_2_ on bituminous coal.

Figure 5 shows the adsorption isotherms of CH_4_ and N_2_ in the molecular structure model of bituminous coal when the temperature is 293 K, 303 K, 313 K, 323 K, and 333 K, and the pressure range is 0.01–10 MPa. The nonlinear form of the Langmuir adsorption isotherm model was used to fit the adsorption equilibrium data of CH_4_ and N_2_, and the fitting curves are shown in Fig. 5. The results show that when the adsorption temperature is 293 K, 303 K, 313 K, 323 K, 333 K, the square R^2^ of the correlation coefficient of CH_4_ adsorption isotherm fitting curve is 0.9963, 0.9974, 0.9966, 0.9971, 0.9972, respectively. The R^2^ of N_2_ was 0.9979, 0.9992, 0.9974, 0.9994, 0.9991, respectively. It can be seen that the Langmuir adsorption model can well describe the adsorption of the two gases by the molecular structure model of bituminous coal.

As can be seen from Fig. 5, the adsorption capacity of CH_4_ is significantly higher than that of N_2_ under the pressure of 1 MPa. When the pressure increased from 0.01 mpa to 1 MPa, the adsorption capacity of the two gases increased rapidly, and CH_4_ increased the fastest. In the process of pressure increasing from 1 to 8 MPa, the increased rate of adsorption capacity of the two gases showed an obvious downward trend. When the pressure increased to 8 MPa, the adsorption capacity gradually tended to be flat. When the pressure is constant, the adsorption capacity of the two gases decreases gradually with the increase of temperature. The increase in temperature is not conducive to the adsorption of gas, so the adsorption process of gas is exothermic. Under isothermal and isobaric conditions, the adsorption capacity of the two gases in bituminous coal is CH_4_ > N_2_. This is because the amount of gas adsorbed decreases with the increase of molecular dynamics diameter, and the molecular dynamics diameter of N_2_ is larger than that of CH_4_, which makes the adsorption of N_2_ by coal weaker than that of CH_4_.

### Effect of temperature on methane desorption

**Figure 6:**
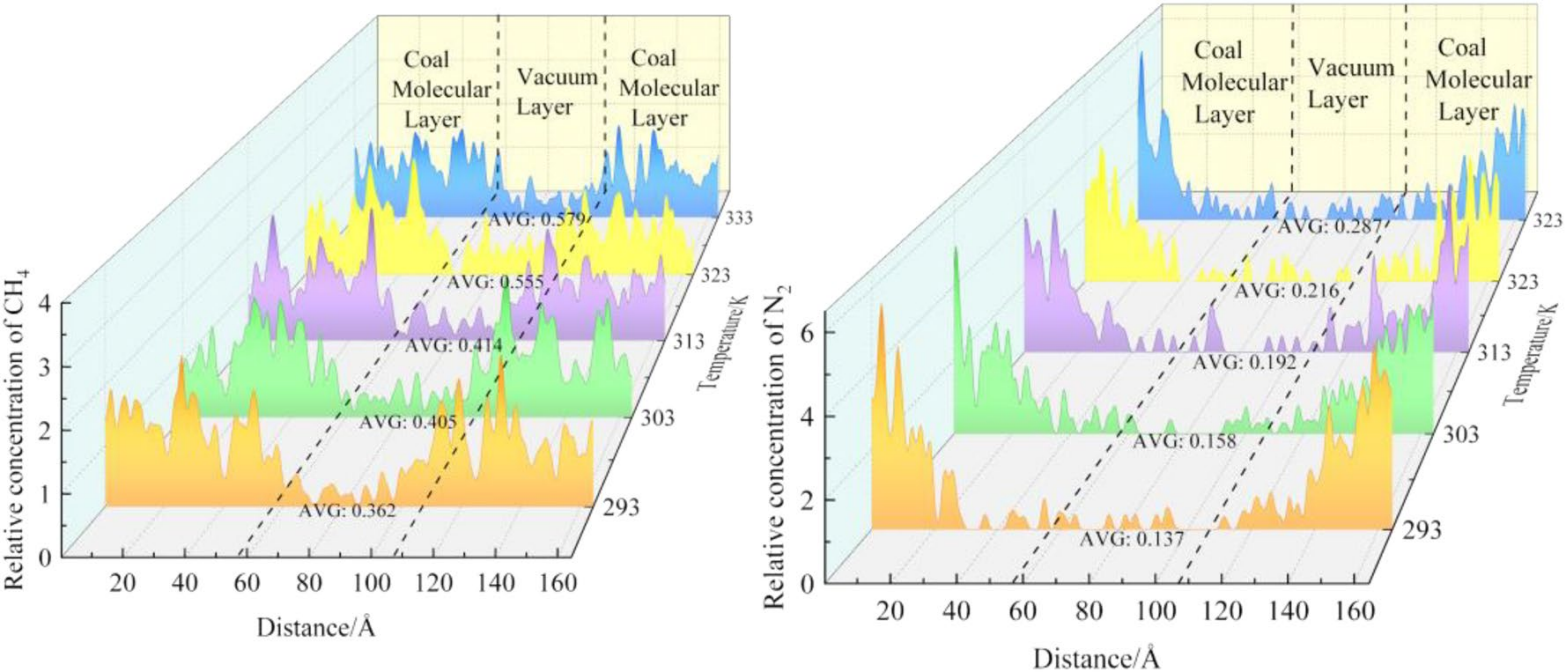
Concentration distribution of CH_4_ and N_2_ at different temperatures.

In this paper, the effect of N_2_ at different temperatures on the determination of CH_4_ in coal is studied from the microscopic point of view. The average relative concentration of the two systems after kinetic optimization is analyzed by the Forcite module. After N_2_ injection, the average relative concentration distribution of the two types of gases in coal molecules is shown in Fig. 6. Regions 0–57 Å and 107–164 Å are the molecular layers of coal, and region 57–107 Å is the vacuum layer. As can be seen from Fig. 6, both types of gases diffuse into the vacuum layer. The increase of CH_4_ molecules in the vacuum layer is the most, and the increase of N_2_ molecules is the least. In the range of 293–333 K, the mean relative concentration of CH_4_ increased by 59.94% after N_2_ injection. At the same temperature, the concentration of CH_4_ in the vacuum layer is 62.15%(293 K), 60.97%(303 K), 53.62%(313 K), 61.08%(323 K), 50.43% (333 K) higher than that of N_2_.

**Figure 7:**
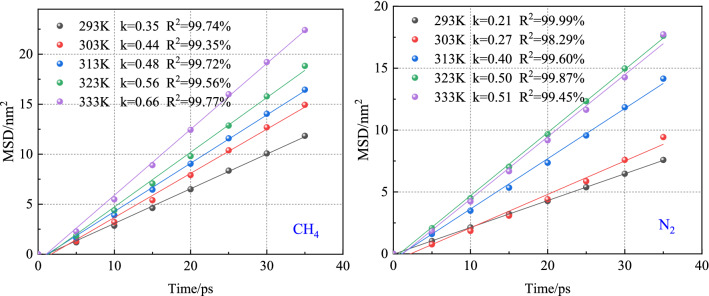
MSD curves of CH_4_ and N_2_ at different temperatures.

As shown in Fig. 7, the root means the square displacement of gas in the CH_4_-N_2_ system increases with the increase of simulation time. At the same time, the MSD of gas in the system increases with the increase of temperature. After N_2_ is injected into the coal, the CH_4_ adsorbed on the coal molecules under the influence of pressure gradient is desorbed out. On the contrary, due to a large number of free N_2_ molecules in the vacuum layer, the concentration of N_2_ in the vacuum layer increases beyond that in the coal molecular layer, and part of N_2_ will diffuse to the coal molecular layer and be adsorbed by the coal molecules. Therefore, what actually happens in coal is that N_2_ resolves CH_4_ by diffusion. At the same temperature, the overall relationship between the root mean square displacement of the two gases is CH_4_ > N_2_.

**Figure 8:**
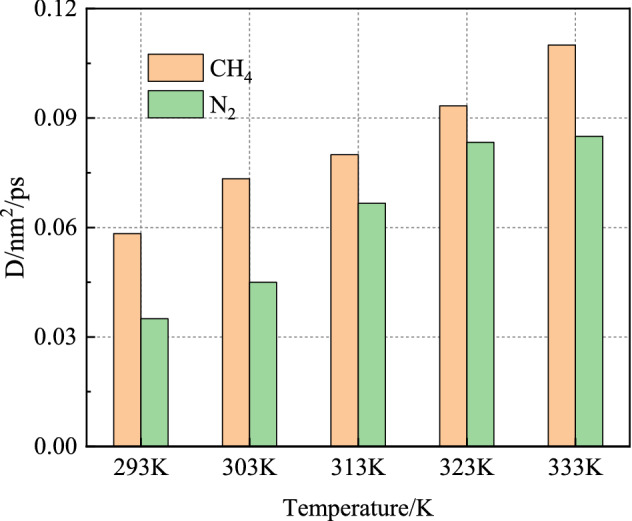
Diffusion coefficients of CH_4_ and N_2_ at different temperatures.

As shown in Fig. 8, the diffusion coefficients of CH_4_ increased with the increase of N_2_ injection temperature, indicating that the molecular activity in the system increased with the increase of temperature. In the range of 293–333 K, the diffusion coefficient of CH_4_ increases from 0.06 to 0.11 nm^2^/ps. The diffusion coefficient of N_2_ increases from 0.03 to 0.08 nm^2^/ps. At the same temperature, the diffusion coefficient of CH_4_ is always greater than that of N_2_. It can be seen that the CH_4_ molecule is more active in the N_2_-CH_4_ system.

### Effect of pressure on methane desorption

**Figure 9:**
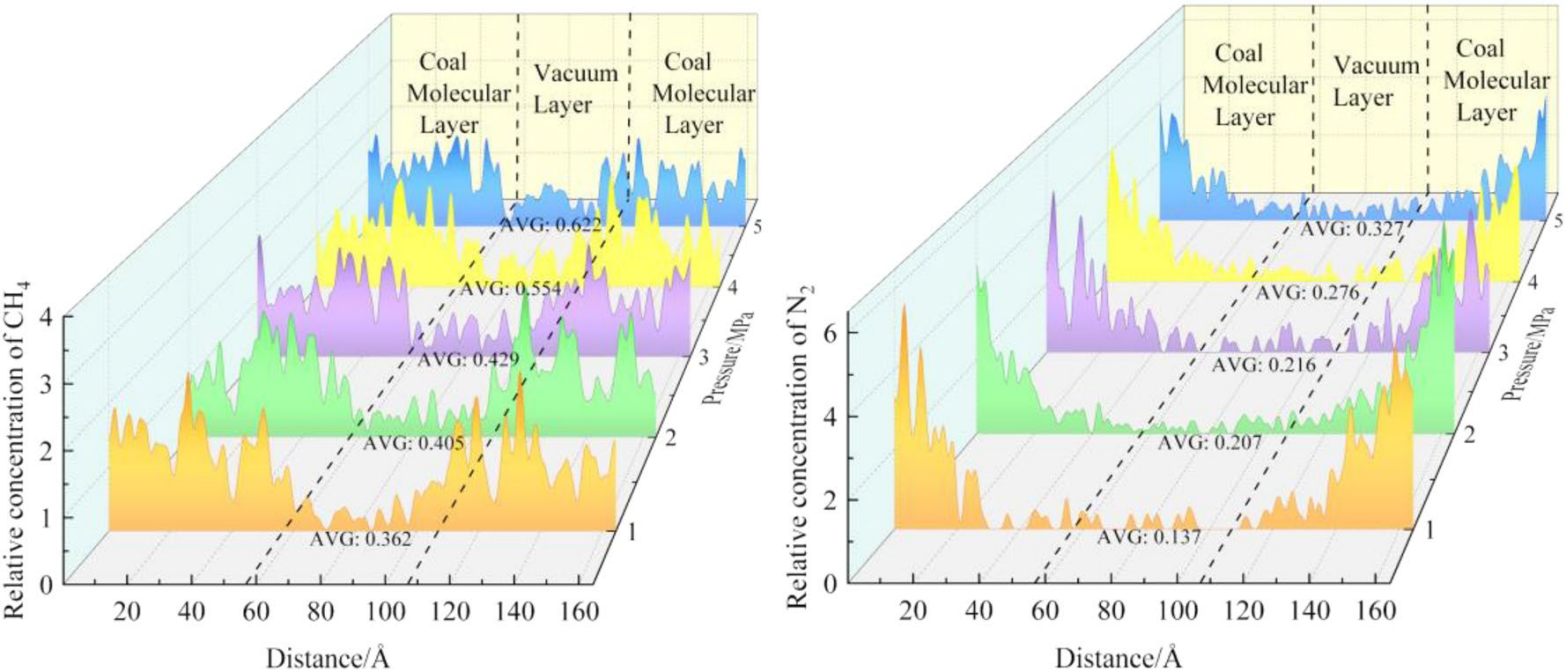
Concentration distribution of CH_4_ and N_2_ under different pressures.

The average relative concentration distribution of the two types of gases in coal molecules after N_2_ injection at different pressures is shown in Fig. 9. As can be seen from Fig. 9, both types of gases diffuse into the vacuum layer. The increase of CH_4_ molecules in vacuum layer is the most, and the increase of N_2_ molecules is the least. The concentration in the vacuum layer increases with the increase of pressure. In the range of 1–5 MPa, the mean relative concentration of CH_4_ increased by 71.82% after N_2_ injection. Under the same pressure, the concentration of CH_4_ in the vacuum layer is 62.15%(1 MPa), 48.88%(2 MPa), 49.65%(3 MPa), 50.18%(4 MPa), and 47.23% (5 MPa) higher than that of N_2_.

**Figure 10:**
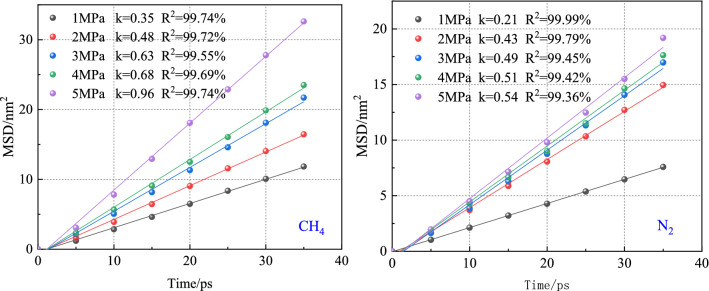
MSD curves of CH_4_ and N_2_ under different pressures.

Figure 10 shows the root means square displacements of CH_4_ and N_2_ at different pressures. In the CH_4_-N_2_ system, the root means square displacements of gases increased with the increase of the simulated pressure. Under the same pressure, the overall relationship between the root means square displacement of the two gases is CH_4_ > N_2_.

**Figure 11:**
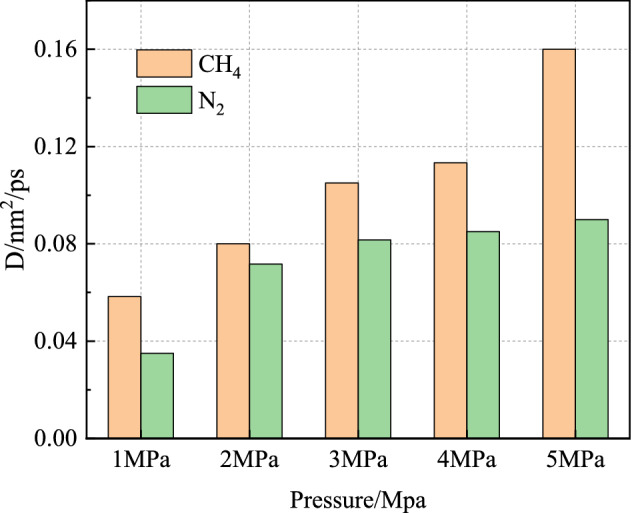
Diffusion coefficients of CH_4_ and N_2_ under different pressures.

Figure 11 shows the relationship between the CH_4_ diffusion coefficient and N_2_ injection pressure. It can be seen from the figure that the diffusion coefficient of both gases increases with the increase of pressure, indicating that the molecular activity in the system increases with the increase of pressure. In the range of 1–5 MPa, the diffusion coefficient of CH_4_ increases from 0.06 to 0.16 nm^2^/ps. The diffusion coefficient of N_2_ increases from 0.03 to 0.09 nm^2^/ps. The diffusion coefficient of CH_4_ is always greater than that of N_2_ under the same pressure. It can be seen that the CH_4_ molecule is more active than N_2_ in the N_2_-CH_4_ system. Since the diffusion coefficient of CH_4_ is higher than that of N_2_, the concentration of CH_4_ diffusing into the vacuum layer is also higher than that of N_2_.

### Effect of water content on methane desorption

**Figure 12:**
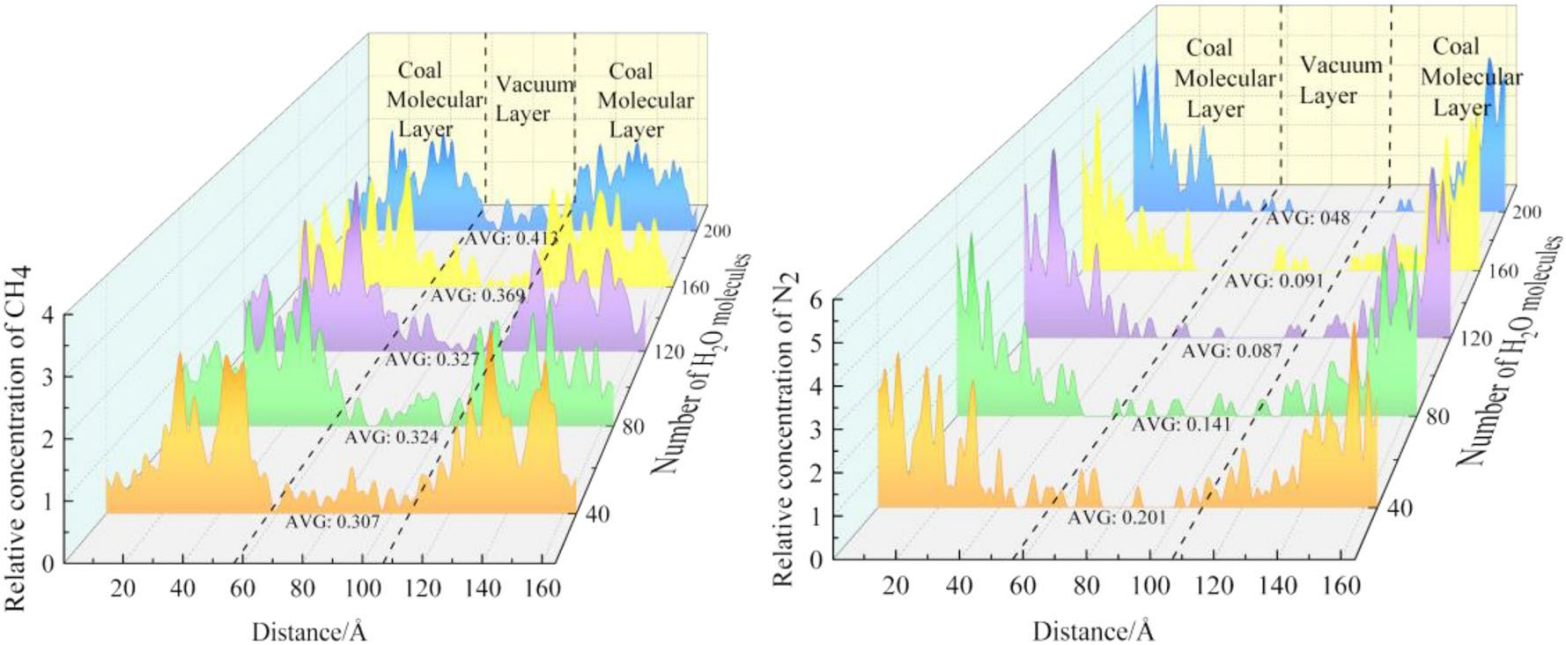
Concentration distribution of CH_4_ and N_2_ under different water content.

As shown in Fig. 12, the relative concentration of CH_4_ in the vacuum layer gradually increases with the increase of water content. This indicates that the water in coal promotes the desorption of CH_4_. This is because the force between coal and methane molecules is greater than that between water molecules, but water molecules are polar molecules, and there is hydrogen bonding between coal and water molecules, so the estimated force between coal and water molecules is far greater than that between coal and methane molecules. Therefore, from the perspective of intermolecular forces, H_2_O is in a dominant competitive position. Because the interaction between coal and water molecules is far greater than that between coal and methane molecules, when the three molecules coexist, water molecules and methane molecules compete for adsorption on the surface of coal, and water molecules will replace some of them to adsorb methane.

### Discussion on the action mechanism of gas molecules

By comparing the effects of injection pressure and temperature on methane resolution, it can be seen that the average relative concentration of CH_4_ increases by 59.94% at 1 MPa and 293–333 K. The diffusion coefficient increased from 0.06 to 0.11 nm^2^/ps. The average relative concentration of CH_4_ at 1–5 MPa and 293 K increased by 71.82% after N_2_ injection. The diffusion line increased by 0.10 nm^2^/ps. It shows that the influence of injection pressure on methane desorption is greater than that of temperature in the simulated range. Compared with the injection of high-temperature nitrogen to desorption coal seam methane, the diffusion effect of high-pressure nitrogen flowing in the coal body is more significant. The higher the injection pressure of nitrogen, the better the effect of N_2_ promoting CH_4_ desorption.

According to the above simulation results, the concentration of desorbed CH_4_ changes with the pressure and temperature of N_2_ injection. With the injection of N_2_, the pore pressure in the coal increases, and the diffusion velocity of CH_4_ in the coal accelerates accordingly. The adsorbed CH_4_ in the coal also begins to desorption and flow out with N_2_ airflow. The increase of gas injection pressure accelerates the seepage rate, improves the pore pressure in the coal body, and promotes the increase of adsorbed CH_4_ desorption amount. Therefore, the desorbed CH_4_ rises with the increase of gas injection pressure. Within the same temperature, the higher the pressure is, the greater the CH_4_ analytical amount is, and the more obvious the promoting effect is. After the injected N_2_ enters the coal, the pressure in the coal increases, and the partial pressure of N_2_ also shows an upward trend. When CH_4_ does not desorption, the partial pressure of CH_4_ remains unchanged. However, after N_2_ injection, there is an additional gas and partial pressure in the system, so the adsorbed CH_4_ will desorption and become a free state. In this simulation, CH_4_ in the coal body is resolved with the continuous injection of N_2_, and the partial pressure of CH_4_ decreases, which intensifies the desorption of adsorbed CH_4_ in the coal body. When the partial pressure of N_2_ promotes the desorption of CH_4_, a part of N_2_ will also be adsorbed into the coal, which shows that N_2_ is adsorbed by the coal while CH_4_ is replaced. It can be seen that the higher the N_2_ injection pressure and temperature, the more methane desorption, and N_2_ can replace some adsorbed CH_4_ through competitive adsorption with CH_4_. The simulation results are consistent with Zhou’s experimental results^26^.

Due to the decrease of partial pressure, CH_4_ in coal will desorption from the pores of the matrix. After desorption of CH_4_ molecules adsorbed in coal, its main form of movement is diffusion movement under the action of the concentration gradient. In this simulation, after N_2_ injection, CH_4_ in the vacuum layer was diluted, which resulted in a concentration difference between the coal molecular layer and the vacuum layer, and directional gas diffusion continued. Not only does CH_4_ diffuse from the coal molecular layer to the vacuum layer, but also N_2_ diffuses into the vacuum layer under the effect of concentration difference. N_2_ promotes the desorption of CH_4_ by diffusion. This is also one of the mechanisms by which N_2_ injection promotes CH_4_ desorption.

## Conclusions

In this paper, the desorption of CH_4_ by N_2_ injection was studied by using GCMC and MD methods. The results show that:

1. The adsorption isotherms of CH_4_ and N_2_ in the molecular structure model of bituminous coal are in good agreement with the Langmuir adsorption isotherm model. The adsorption capacity of the two gases in the bituminous coal structure model is CH_4_ > N_2_. Temperature is negatively correlated with the adsorption amount.
2. In the range of 293–333 K, the average relative concentration of CH_4_ in the vacuum layer increased by 59.94% after N_2_ injection. In the range of 1–5 MPa, the average relative concentration of CH_4_ increased by 71.82% after N_2_ injection. The higher the N_2_ injection pressure and temperature, the more methane desorption, and N_2_ can replace some adsorbed CH_4_ through competitive adsorption with CH_4_. The activity of the CH_4_ molecule is higher in N_2_–CH_4_ system.
3. The relative concentration of CH_4_ in the vacuum layer gradually increases with the increase of water content. This indicates that the water in coal promotes the desorption of CH_4_. The results of this article provide theoretical support for methane extraction technology in goaf. Since this study only carried out a molecular simulation on the bituminous coal model, the research scope will be expanded in the future to further improve the research results.

## Data Availability

The datasets used and/or analysed during the current study available from the corresponding author on reasonable request.
